# Liver organoids: from basic research to therapeutic applications

**DOI:** 10.1136/gutjnl-2019-319256

**Published:** 2019-07-12

**Authors:** Nicole Prior, Patricia Inacio, Meritxell Huch

**Affiliations:** 1 Wellcome Trust-Cancer Research UK Gurdon Institute, University of Cambridge, Cambridge, UK; 2 Max Planck Institute of Molecular Cell Biology and Genetics, Dresden, Germany

**Keywords:** liver, organoid, personalised medicine, disease modelling

## Abstract

Organoid cultures have emerged as an alternative in vitro system to recapitulate tissues in a dish. While mouse models and cell lines have furthered our understanding of liver biology and associated diseases, they suffer in replicating key aspects of human liver tissue, in particular its complex architecture and metabolic functions. Liver organoids have now been established for multiple species from induced pluripotent stem cells, embryonic stem cells, hepatoblasts and adult tissue-derived cells. These represent a promising addition to our toolbox to gain a deeper understanding of this complex organ. In this perspective we will review the advances in the liver organoid field, its limitations and potential for biomedical applications.

## Introduction

### What are organoids? How can organoids benefit research?

The study of human organ development and diseased states is hampered by the inaccessibility of human samples in vivo and intrinsic differences between animal models and human biology. Advances in three-dimensional (3D) cell culture techniques facilitated by a deeper understanding of extracellular matrix (ECM) biology combined with greater knowledge about signalling pathway regulation of stem cell niches and differentiation programmes have enabled the establishment of organoid culture systems. The term ‘organoid’ has previously been used to refer to a range of 3D culture systems which resemble the modelled organ to varying extents. Here we subscribe to the organoid definition coined by Lancaster and Knoblich^1^ and Huch and Koo^2^ to define an organoid as an in vitro 3D cellular cluster derived from tissue-resident stem/progenitor cells, embryonic stem cells (ESCs) or induced pluripotent stem cells (iPSCs) capable of self-renewal and self-organisation that recapitulates the functionality of the tissue of origin. Organoids were named ‘Method of the year 2017’ by *Nature Methods*,^3^ reflecting the excitement and promise of this rapidly expanding field to provide new experimentally tractable, physiologically relevant models of organ development, human pathologies and paving the way for therapeutic applications.

In the body, cells reside in complex microenvironments and are subject to numerous signalling interactions, including those from soluble factors, mechanical cues and the ECM. These interactions are key in establishing, maintaining and regulating cellular phenotypes and functions. It is now broadly accepted that cells cultured in 3D more closely resemble architectural and functional properties of in vivo tissues compared with cells cultured with two-dimensional (2D) techniques. One reason for this is the generation of cell–cell or cell–ECM interactions in all three dimensions, while in 2D monolayer cultures interactions are limited to the horizontal plane. Cells within a tissue are often exposed to concentration gradients of signalling effector molecules, nutrients and waste products; this is mimicked to an extent in 3D culture systems with the cells at the centre of an aggregate/organoid having less access to factors in the culture medium. Conversely, in 2D monolayers cells are exposed to a uniform concentration of factors due to direct contact with the culture medium. The establishment of more physiological, biochemical and biomechanical microenvironments using 3D techniques can affect cell proliferation, differentiation, morphogenesis, cell migration, mechanoresponses and cell survival.^4^ In terms of therapeutic applications, this may, in part, explain the failure of 2D cell culture systems to recapitulate drug screening outcomes as seen in vivo.^5^ Organoids represent a promising model system to bridge the gap between 2D cultures and in vivo mouse/human models. Organoids are more physiologically relevant than 2D culture models, while providing a reductionist model of in vivo biology in which it is possible to manipulate signalling pathways and perform genome editing.

**Figure 1:**
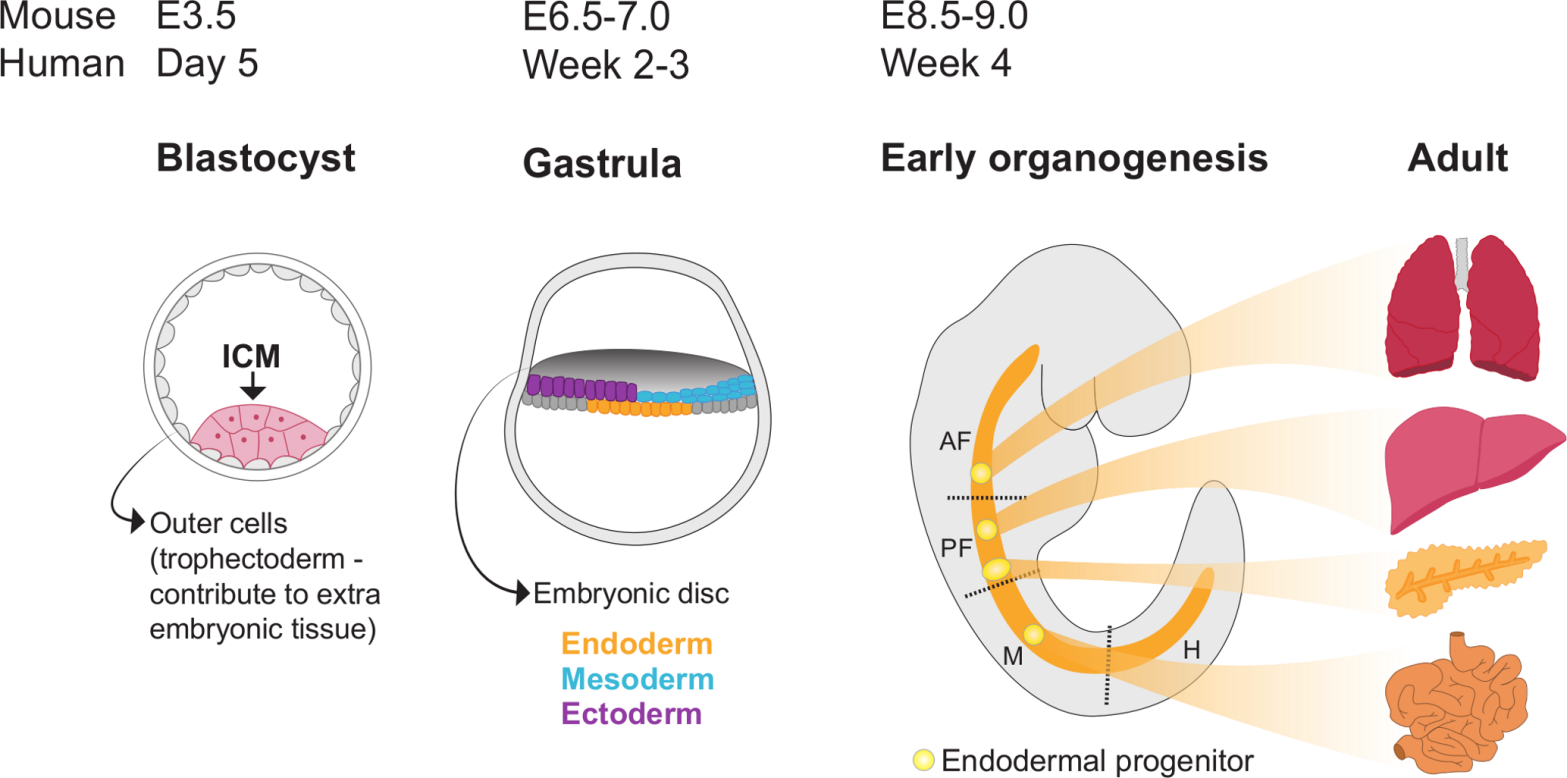
Organogenesis and stages for organoid progenitor isolation. Schematic depicting key stages of organogenesis timings in mice and humans. Following fertilisation and cleavage of the embryo, the blastocyst is formed in which cells segregate into the outer layer and the inner cell mass (ICM). Cells of the ICM are pluripotent and can be isolated to generate embryonic stem cells. The next key developmental milestone is gastrulation, a process whereby cells derived from the ICM undergo dynamic cell movements and rearrange to form the three germ layers: endoderm, mesoderm and ectoderm. Here we depict a human gastrula which develops as an embryonic disc (note: gastrulation in mice occurs as an egg cylinder). As development progresses, progenitors within each germ layer become specified to give rise to specific tissues and organs. The identities of the progenitors are influenced by their anterior-posterior and dorsal-ventral positions in the embryo. The endoderm becomes patterned along the anterior-posterior axis into the anterior foregut (AF), posterior foregut (PF), midgut (M) and hindgut (H). Illustrated here are a selection of organs that derive from the different endodermal domains: AF—lungs; PF—liver and pancreas; M—small intestine. The hindgut gives rise to more posterior tissues such as the colon. Organoids can be derived from tissue-resident progenitors isolated at both organogenesis stages and from adult tissues.

Initiation of organoid culture requires the isolation of stem/progenitor cells, either pluripotent stem cells (PSCs) or tissue-resident stem/progenitor/differentiated cells isolated from embryonic stages or adult tissues (figure 1). The cells of origin for PSC-derived organoids are ESCs or iPSCs, which are then cultured in media supplemented with growth factors in order to mimic the signals that cells are exposed to during embryonic patterning to give rise to the specific tissue.

During development, a single totipotent cell, the zygote, which can form extraembryonic and embryonic tissues, proliferates and gives rise to progeny that overtime becomes increasingly lineage-restricted. At the blastocyst stage the outer cells are committed to extraembryonic fates, while cells of the inner cell mass (ICM) are pluripotent and are competent to form all tissues of the embryo proper; it is these pluripotent ICM cells that are isolated to obtain ESCs.^6^ The next key developmental process is gastrulation, where cells derived from the ICM undergo extensive cell mixing and morphogenetic movements mediated by signalling factors such as Wnt, fibroblast growth factor (FGF) and transforming growth factor-beta ligands to activate transcriptional programmes and subsequent differentiation into the three germ layers: endoderm, mesoderm and ectoderm. Within the three germ layers progenitor cells become specified to form primordial organ structures, which give rise to all tissues and organs of the body. The identity of these progenitors is regulated by signalling gradients of morphogens that are established along the anterior-posterior and dorsal-ventral axes of the developing embryo. The GI tract derives from the endodermal germ layer, and depending on the location of the endodermal progenitors they may generate tissues with anterior identities such as the lungs, or with more posterior identities such as the small intestine. This is due to anterior-posterior gradients of signalling factors, including Wnt, retinoic acid, bone morphogenetic protein (BMP) and FGFs; dysregulation of these gradients can have drastic consequences, for example overactivation of the Wnt pathway causes more anterior endodermal progenitors to adopt hindgut fates, leading to the failure of liver formation.^7^ Likewise, the fate of PSCs in culture can be influenced by the activation and inhibition of developmental signalling pathways to direct the stepwise in vitro differentiation of progenitors towards specific organ identities.

Organoids generated from tissue-resident stem/progenitor cells require culture conditions that resemble the stem cell niche during physiological tissue self-renewal or during damage repair, rather than recapitulation of developmental processes. In addition to use of carefully composed culture media, organoid systems require a specialised physical environment, which commonly involves culturing multipotent progenitor(s) in suspension, on an air–liquid interface or embedded in a suitable ECM such as Matrigel. Under these conditions, the multipotent progenitor(s) follows intrinsic developmental or homeostatic/repair programmes to proliferate and self-organise into 3D organoid structures.

### History of organoids

The rapid advances of the organoid field over the last 10 years are built on decades of work to gain greater insight into PSCs, self-organisation of dissociated tissues and ECM biology.^8^ For example, Bissel and colleagues^9^ demonstrated that interactions with the ECM could improve hepatocellular function of rat hepatocytes and regulate the growth and differentiation of mammary gland epithelia which, when embedded in ECM hydrogels, could develop tubules and ducts.^10 11^ Another demonstration of the importance of recapitulating cell–cell and cell–ECM interactions was the coculture of gastric epithelial cells and fibroblasts in an ECM hydrogel with an air–liquid interface. This method enabled the short-term culture of stomach tissue and facilitated the generation of differentiated gastric surface mucous cells.^12^ These early studies showed that 3D culture systems had the potential to support morphological rearrangements and differentiation of tissues but were limited to short-term cultures which could not self-renew.

Seminal work from the lab of Hans Clevers demonstrated that a single *leucine-rich repeat-containing G-protein-coupled receptor 5* (*Lgr5*) positive adult stem cell isolated from mouse intestinal tissue could form a self-renewing culture that recapitulated the intestinal crypt-villus architecture and cell composition, including mature functional cell types.^13^ The isolated stem cell was embedded in Matrigel as ECM and cultured in media supplemented with growth factors based on the endogenous intestinal stem cell niche. A key finding from this report was the inclusion in the culture media of R-spondin, an LGR4/5/6 ligand that upregulates Wnt signalling, which helps to maintain stem cell populations. Since this initial finding, culture of human intestinal organoids has been achieved,^14^ and R-spondin containing media has been further optimised to support the generation of organoids from other organs with *Lgr5+* progenitor cells, including the colon,^14^ stomach^15^ and liver,^16^among others.

Simultaneous to the development of organoids derived from tissue-resident progenitors/cells, ESCs were shown to generate cortical tissues when differentiated as 3D aggregates,^17^ which laid the foundation for the generation of cerebral organoids that could model aspects of human brain development in vitro.^18^ Since these reports, organoids from both PSCs as well as tissue-resident stem cells have been used to model many organs derived from the endoderm, mesoderm and ectoderm as reviewed in ref 19. Briefly we present a non-exhaustive list of organoid systems to date: organoids derived from the endoderm: thyroid,^20 21^ lung,^22–24^ stomach,^15 25 26^ liver,^16 27–29^ pancreas,^30–33^ small intestine^13 34^ and colon^14 35^; mesoderm-derived organoids: kidney,^36^ bone,^37^ fallopian tube^38^ and endometrium^39 40^; and ectoderm-derived organoids: mammary gland,^41 42^ retinal,^43–45^ brain,^18 46 47^ inner ear^48^ and salivary gland.^49^ In this perspective we will focus on the establishment of liver organoids, how they can be used to study liver development and disease, and their therapeutic potential.

### Liver development, homeostasis and regeneration

The majority of the liver is composed of epithelial cells (hepatocytes and cholangiocytes) that work together with stromal, endothelial and mesenchymal cells to perform crucial metabolic, exocrine and endocrine functions for body homeostasis. Our knowledge of liver development stems greatly from mouse studies, which despite intrinsic biological differences from human can guide researchers’ efforts to direct differentiation of liver progenitors and understand human development. During organogenesis liver embryonic progenitor cells (known as hepatoblasts) are specified from the posterior foregut endoderm. In response to signalling factors secreted by the surrounding mesenchyme, such as FGF, BMP, hepatocyte growth factor (HGF) and Wnt, hepatoblasts undergo cell shape changes, proliferate and migrate into the adjacent mesoderm to form the liver bud.^50^ During the course of liver bud outgrowth and the establishment of the lobes, hepatoblasts become lineage-committed in order to give rise to hepatocytes and cholangiocytes.^51^ Indeed, we recently reported that a single *Lgr5+* hepatoblast can generate both hepatocytes and cholangiocytes, demonstrating the bipotency of hepatoblasts in vivo.^52^ The fate of hepatoblasts is influenced by local signalling: subsets of hepatoblasts that are exposed to signals near the portal mesenchyme generate cholangiocytes, while hepatoblasts that are located further from the portal veins respond to signals from closely associated haematopoietic cells and give rise to hepatocytes.

**Figure 2:**
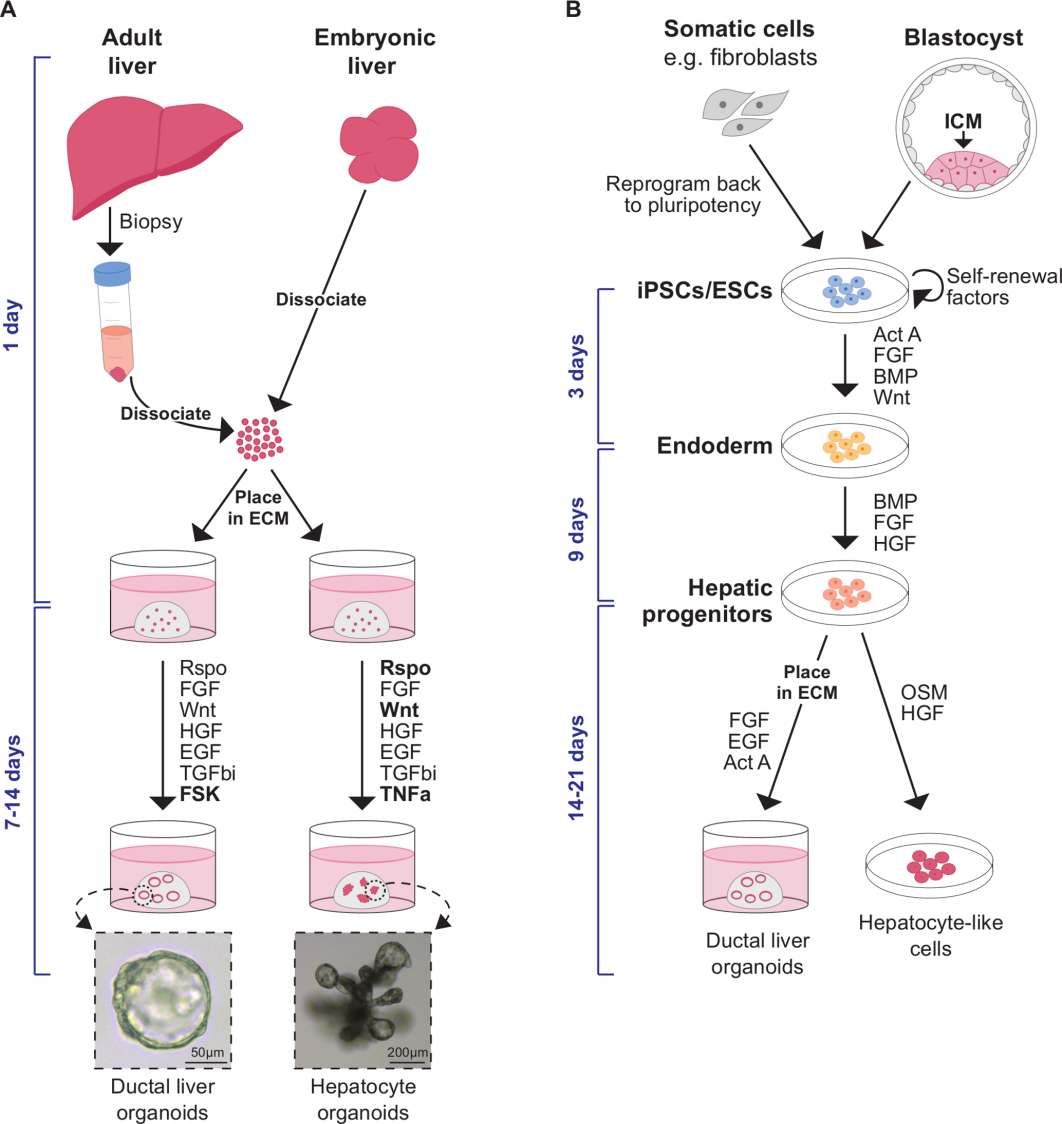
Liver organoids can be derived from various cells of origin by regulating signalling pathways during in vitro culture. (A) Liver organoids can be formed from tissue-resident cells isolated from biopsies of adult tissues or from embryonic stages during organogenesis. Hepatoblasts (the bipotent embryonic progenitors in vivo which give rise to ductal cells and hepatocytes) can be placed in Matrigel as ECM and generate ductal or hepatocyte organoids depending on the growth factors supplemented in the culture medium. (Bright-field images of mouse embryonic ductal and hepatocyte organoids taken from Prior *et al*.^52^) Signalling pathways which are typically modulated to enable organoid formation are listed; the pathways which are essential for different types of liver organoids are in bold. Formation of ductal or hepatocyte organoids from adult tissues requires the isolation of appropriate cells of origin. In order to generate ductal hepatic organoids from adult tissues, ductal fragments or ductal cells can be placed in Matrigel with the optimised media. Formation of adult hepatocyte organoids requires the isolation of mature hepatocytes. (B) Liver organoids can also be generated from pluripotent stem cells (iPSCs and ESCs), usually by a three-stage differentiation process that recapitulates the signalling programmes active during development. iPSCs/ESCs are first directed towards an endodermal fate by exposure to Act A and Wnt. These endoderm cells then progress to a hepatic fate following induction of HGF and FGF signalling. These hepatic progenitors are hepatoblast-like cells. The hepatic progenitors can form hepatocyte-like cells in response to OSM signalling. Conversely, by placing the hepatic progenitors in ECM and modulating FGF, EGF and Act A signalling, ductal organoids can be generated. Act A, Activin A; BMP, bone morphogenetic protein; ECM, extracellular matrix; EGF, epidermal growth factor; ESCs, embryonic stem cells; FGF, fibroblast growth factor; FSK, forskolin; HGF, hepatocyte growth factor; ICM, inner cell mass; iPSCs, induced pluripotent stem cells; OSM, Oncostatin M; TGFbi, transforming growth factor beta inhibitor; TNFa, tumour necrosis factor-alpha.

To support normal functions, the adult liver must be maintained during homeostasis. In contrast to other endodermal organs such as the intestine that self-renew every 3–5 days, the liver has a much slower cellular turnover (in mice, approximately every 60 and 150 days for cholangiocytes and hepatocytes, respectively^53^). Homeostatic epithelial maintenance occurs primarily through the self-duplication of mature cells.^54 55^ Despite a low cellular turnover, when challenged, the liver has a remarkable ability to regenerate, although repeated damage to the tissue can result in impairment of liver function and fibrosis, as reviewed in ref 56. Upon partial hepatectomy (surgical resection of up to two-thirds of the liver), the remaining healthy mature hepatocytes respond to injury-induced regenerative signals such as tumour necrosis factor-alpha (TNFa) and interleukin-6 to proliferate and undergo hyperplasia in order to restore tissue mass within a week.^57 58^ Understanding of this phenomenon has been taken into the clinic and helped to facilitate live donor transplants and tumour resections. However, on toxin-mediated damage (eg, viruses and alcohol) or due to chronic liver pathologies such as non-alcoholic fatty liver disease (NAFLD), hepatocytes become impaired and are unable to undergo the mass proliferative response seen following partial hepatectomy. Incredibly, even when hepatocyte proliferation is compromised, the liver is still capable of regenerating itself. In this case, there is a ductular reaction in which ductal cells become activated and start to proliferate, repopulating the liver.^59–62^ Understandably, there has been a large effort to establish faithful in vitro liver models from PSCs and tissue-resident stem/progenitor/differentiated cells to gain insights into liver biology and diseases and into regenerative mechanisms in general (figure 2).

### Embryonic liver organoids as a tool to understand liver development

With the aim of replicating liver development in vitro, several groups have succeeded in differentiating human iPSCs into hepatocyte-like cells following a stepwise differentiation protocol based on several chemical inhibitors.^63^ An alternative approach was pursued by the Suzuki and Hui labs, which forced the expression of pioneer liver transcription factors (*HNF4a* and *FOXA1,2,3*)^64^ or (*HNF4a*, *GATA4* and *HNF1B*)^65^ to induce the direct differentiation of iPSCs into hepatocyte-like cells in vitro. However, these early differentiation approaches were performed in 2D and lacked the 3D information required to form hepatic tissue in vitro. The first attempt to generate 3D liver tissue in culture that recapitulated embryonic liver features was the establishment of embryonic liver bud cultures by Takebe and colleagues,^66^ in which human iPSC-derived hepatocytes were cultured with mesenchymal stem cells and umbilical cord cells. The resulting liver bud organoids consisted of proliferating hepatoblasts and associated cells that, on transplantation into different mouse sites, developed into hepatic tissue exhibiting mature hepatic features.^66^ This system has since been refined so that the hepatic endoderm, mesenchymal and endothelial progenitors are all derived from iPSCs.^67^ Human iPSCs can also be directed via the exposure to specific signalling pathways to form cholangiocyte organoids.^68^ Due to the careful modulation of culture media necessary to direct PSCs to a specific lineage, a major challenge that organoid models need to resolve is successful coculture of multiple cell types with different media requirements. Recently, hepatobiliary structures containing both hepatocyte and cholangiocyte cells have been derived from human iPSCs,^69 70^ although these structures do not self-renew. These reports may facilitate refinement of culture media to soon generate self-organising, self-renewing hepatobiliary organoids.

One limitation of iPSC-derived organoids for clinical use is the concern regarding genomic instability due to exposure to reprogramming factors.^71^ To negate this concern, organoids can be derived from embryonic or adult tissue-resident stem cells. We recently described the isolation of bipotent *Lgr5*+ embryonic hepatoblasts which retain the capacity to form either hepatocyte or cholangiocyte organoids depending on the culture medium used.^52^ Similarly, the establishment of hepatocyte organoids derived from bulk human embryonic liver tissue from aborted fetuses has been described.^28^ Further optimisation of culture conditions may enable the development of hepatobiliary organoids from these tissue-resident stem progenitors. Hepatoblasts serve as the functional stem cell of the liver during development; however, they are not generally thought to persist postnatally. Therefore, in order to recapitulate liver regeneration and diseased states in vitro, it may be beneficial to initiate cultures using cells isolated from primary adult liver tissue.

### Liver organoids that recapitulate adult tissue and liver regeneration

Early studies by Michalopoulos and colleagues,^72^ in which isolated adult rat hepatocytes and other hepatic cells were placed in roller cultures, led to the formation of tissues resembling features of hepatic architecture; however, these cultures only survived for a short period of time. Self-renewing liver organoids demonstrating genetic stability during long-term culture were reported in 2013. Isolated healthy ducts (or *Lgr5*+ liver cells postdamage induction) self-organised into 3D structures which sustained long-term expansion as adult ductal progenitor cells while retaining the ability to differentiate into functional hepatocyte-like cells in vitro.^16^ The expansion of adult ductal liver organoids was enabled by use of an optimised culture medium. In addition to the epidermal growth factor and Wnt agonists, which had been shown to be important for organoid derivation from *Lgr5*+ intestinal stem cells,^13^ signalling factors important for liver development, HGF and FGF, were supplemented in the culture medium. This protocol was subsequently adapted to generate liver organoids from healthy human primary tissue^27^ and to generate disease models from primary liver cancer (PLC) (discussed below).^73^ Recently, the Nusse and Clevers labs have described the long-term culture of primary mouse hepatocytes,^28 29^ which retain many morphological and functional properties of hepatocytes. The system proposed by Hu *et al* 28 uses increased R-spondin and FGF signalling to produce hepatocyte organoids that have expression profiles similar to those of hepatocytes after partial hepatectomy. The model demonstrated by Peng *et al* 29 was based on observations that during liver injury, liver-resident macrophages secrete high levels of inflammatory cytokines, including TNFa, to aid regeneration. Culture with TNFa indeed served to enhance expansion of hepatocytes.^29^

In addition to liver organoid generation from mouse and human cells, adult ductal organoids have also been generated from rats,^74^ cats^75^ and dogs^76^; for an extended review on liver organoids from other species please see ref 77. Since several liver pathologies progress in a similar manner in cats and dogs as in humans, use of organoids from these species may provide insight to advance human therapies while not being subject to the same level of ethical constraints.

**Figure 3:**
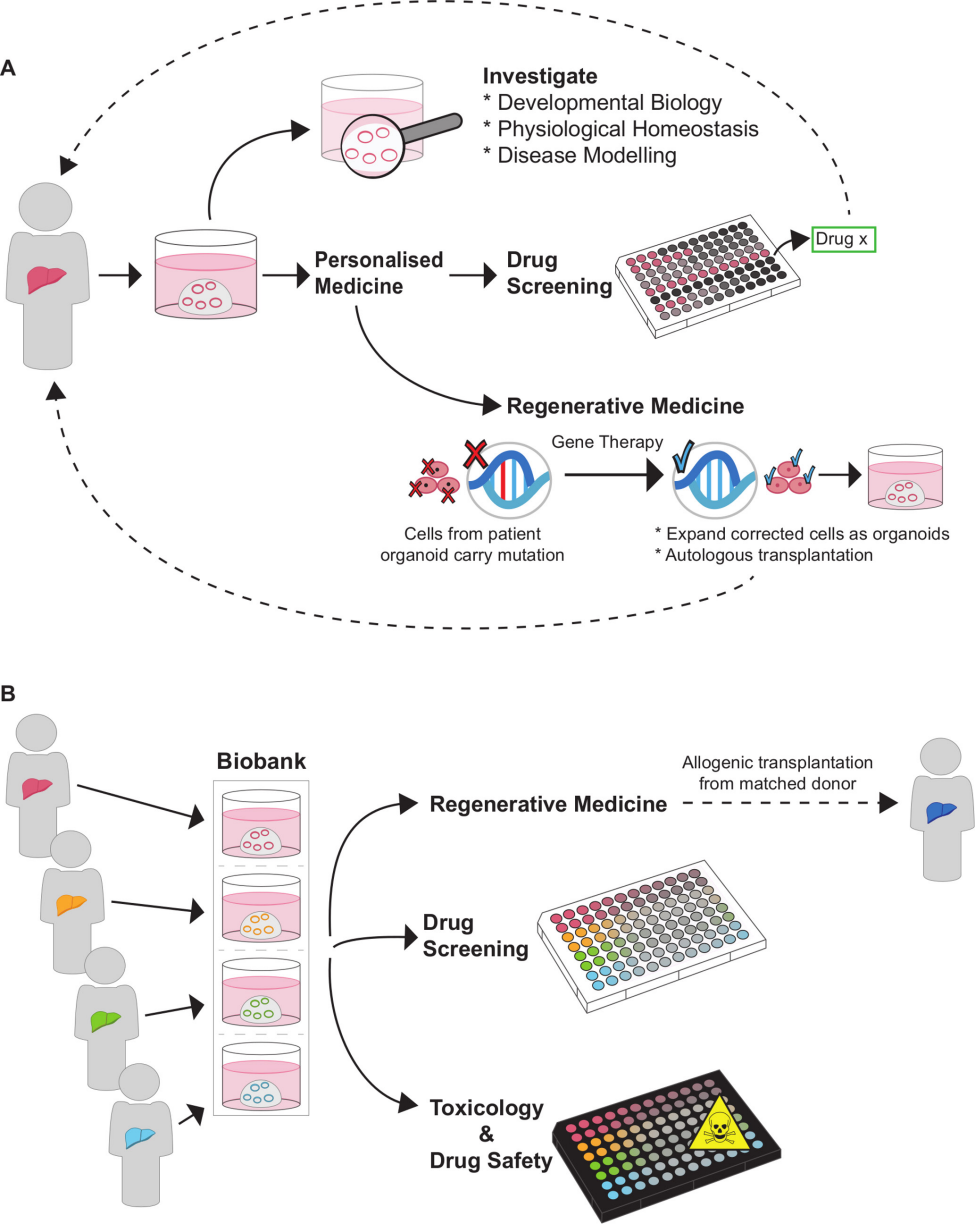
Applications of liver organoids. (A) Organoids derived from healthy donors or patients can be used as a model in basic research to investigate liver development and function in healthy conditions and to dissect the mechanisms of disease. Liver organoids are also a potential bridging tool towards personalised medicine, allowing for patient-specific drug screening and gene therapy. (B) Organoids can be expanded in vitro and cryopreserved enabling the establishment of biobanks. These can be used on a larger scale for regenerative medicine (including transplants), drug screening (patient-derived organoids can help identify drugs that a cohort of patients are most likely to respond to) and toxicology studies for predicting which potential therapies may induce drug-induced liver injury.

These advances in liver organoid technology are providing models for prenatal development, tissue maintenance and pathologies, which are otherwise intractable processes to study in human. Below we discuss the biomedical applications of liver organoids, including their use for disease modelling (of both monogenic and acquired liver diseases), drug screening, toxicology studies and regenerative medicine (figure 3).

### Disease modelling

#### Monogenic liver diseases

Monogenic liver diseases are caused by mutations in single genes. While these are considered ‘rare’ conditions, they account for 10 in every 1000 births.^78^ These represent a heterogeneous group of diseases that, according to the extent of the damage to the parenchyma and/or specific liver expression, can be classified into three groups: (1) conditions associated with predominant liver parenchymal damage, (2) disorders in which liver architecture is near normal, and (3) genetic defects with both hepatic and extrahepatic manifestations.^78^

Within the monogenic diseases with both intrahepatic and extrahepatic manifestations, cystic fibrosis (CF) was the first human monogenic disease modelled with organoids, specifically using human intestinal organoids derived from rectal biopsies of patients with CF.^79^ The disease is caused by mutations in the cystic fibrosis transmembrane conductance regulator (*CFTR*) gene, which codes for a chloride channel normally expressed in epithelial cells of many organs, including on the surface of cholangiocytes and bile duct cells. Mutations in the *CFTR* gene impair cholangiocyte chloride transport, leading to a lack of alkalinisation and subsequent blockage of biliary ducts in the liver.^80^ Dekkers and colleagues^79^ showed that CF intestinal organoids recapitulated the disease in vitro when cultured with an inducer of intracellular cyclic AMP (cAMP), called forskolin, thereby activating CFTR function. While healthy organoids responded to cAMP by importing fluid to the organoid lumen causing it to swell, this response was abolished in CF intestinal organoids. Subsequently, Sampaziotis and colleagues^68^ and Ogawa and colleagues^81^ also showed that cholangiocyte organoids generated from iPSCs of patients with CF carrying the most common mutation in *CFTR* (Δ*F508*) could also be used to model CF in vitro. Similar to the small intestinal organoids, CF cholangiocyte organoids also lacked the ability to swell in response to forskolin.

Within the panoply of monogenic liver disorders, alpha-1-anti-trypsin (A1AT) deficiency, Wilson’s disease and Alagille syndrome are three examples of diseases that affect the liver parenchyma (for an extensive review on monogenic liver diseases, please see ref 78). Organoids derived from adult liver tissue from patients with either A1AT deficiency or Alagille syndrome or dogs with a special form of Wilson’s disease have been successfully developed and recapitulate key aspects of the diseases modelled.^27 76^ Specifically, differentiated liver organoids from patients with A1AT deficiency accumulate protein aggregates, similar to what had been observed in the original biopsy.^27^ Liver organoids derived from biopsies from patients with Alagille syndrome,^27^ or generated by differentiation from iPSCs from patients with Alagille syndrome,^82^ mirrored the in vivo biliary defects that characterise the disease. In addition, liver organoids established from a mouse model of Alagille syndrome (*JAG1* mutants) showed that the mutation caused a delay in the ability of organoids to differentiate into mature cholangiocytes and a failure to form and maintain biliary ducts.^83^ These studies are proof-of-principle that liver organoids recapitulated key features of the disease in vitro and enable further understanding of the disease processes.

#### Acquired liver diseases

Liver organoids were first exploited as tools to model monogenic liver diseases, but recently the 3D structures have been successfully used to model complex acquired liver diseases, including PLC and viral hepatitis.

##### Cancer

PLC, the second most lethal malignancy worldwide, includes a heterogeneous group of tumours with distinct histological features and poor prognosis rates: hepatocellular carcinoma (HCC) represents 80% of all PLCs, followed by cholangiocarcinoma (CC). A combined hepatocellular-cholangiocarcinoma subtype (HCC/CC) accounts for 0.4%–14.2% of all PLCs. PLCs are characterised by a complex and diverse landscape of genetic—including high degree of aneuploidy, DNA copy number variations, somatic mutations—and epigenetic alterations that drive neoplastic transformation and growth,^84^ further supporting the need for patient-tailored therapeutics, also called personalised medicine. The multikinase inhibitor sorafenib^85^ and more recently lenvatinib^86^ are the only two approved first-line therapies for HCC in the USA. Currently, no target therapies are approved for CC. Frequent genetic alterations in PLCs, both HCC and CC, include those in the *TP53* and cell cycle-related genes, such as *CCND1* and *CDKN2A*, and in the chromatin-remodelling genes *ARID1A* and *ARID2.* HCC is further characterised by activating mutations in components of the *WNT*/*CTNNB1* pathway, while CC is frequently altered in the *EGFR* and *KRAS* genes.^84^

For decades, research into PLCs depended on 2D cell culture systems and transgenic mouse models. While these have proven useful to advance our understanding of the disease, both approaches are markedly limited. PLC is genetically heterogeneous, as shown by sequencing studies, a feature that the limited number of existing cells lines fail to reproduce. In fact, the establishment of cell lines represents a bottleneck of the heterogeneous genomic landscape of PLCs, as only the clones with the most beneficial mutations are amenable to in vitro culture. Moreover, 2D cell lines are unable to mimic the histoarchitecture of the tumours. Genetically engineered mouse models (GEMM) have provided insights into the biology of the disease; however, they are time-consuming, costly and cannot recapitulate all the human tumour traits. Patient derived xenografts (PDXs), in which cells from a patient are transplanted into immunocompromised mice, have been established for both (HCC and CC) as alternative approaches to GEMM.^87^ PDXs recapitulate the genetic and histological features of the original tumour,^88^ and if engrafted into humanised mice may help infer the interactions between tumour and immune cells.^89^ The PDX approach has been used with great success to study resistance mechanisms and test different therapeutics.^90 91^ However, while PDXs show great translational potential to direct treatment in a patient-tailored manner, this strategy has several drawbacks: PDXs are not amenable to large-scale drug screens, are costly and can take a considerable amount of time to establish.

By adapting the initial protocol to expand adult liver ductal progenitor cells,^27^ we successfully established primary liver tumour organoids in vitro—called tumouroids—from tumour resections (∼1 cm^3^ tissue) of eight patients with PLC, including its main three subtypes: HCC, CC and HCC/CC.^73^ The cultures expanded long term (~1 year), and preserved the histological architecture, gene expression patterns and genetic alterations seen in the patient tumour tissue of origin. Importantly, the tumouroids maintained tissue-of-origin features over time; global exome sequencing showed that over 90% of the genetic alterations in the patient’s tumour tissue were maintained in the respective tumouroids when in culture for less than 2 months and over 80% after 4 months. Furthermore, tumouroids recapitulated the metastatic potential of the original tumour when transplanted into immunocompromised mice. In addition, comparative gene expression profiling of tumouroids versus healthy liver organoids enabled the identification of novel genes with prognostic value in liver cancer cohorts. These studies demonstrated that liver tumour organoids could be used to identify novel genes with a potentially important role in human liver cancer.

In addition, tumouroids were tested for their potential as a platform for drug screening and validation of candidate therapies^73^; tumouroids responded with varying levels of sensitivity, supporting their use as platform for drug sensitivity testing in a patient-specific manner, opening the doors for precision oncology. The impact of the different sensitive compounds in healthy tissue remains to be tested. This can be achieved using healthy organoids as a platform to dissect the potential side effects of candidate therapies, a promising yet unexplored goal. While the PLC-derived organoids support the broad-ranging translation potential of organoids into the clinics, the limited number of samples used calls for further validation in more patients. Whether tumouroids may also help identify therapies for metastases remains to be investigated. Unfortunately, we were unable to establish tumouroids from very well-differentiated tumours (with less than 5% of proliferating cells), precluding drug testing for less advanced tumours. Whether refining the medium conditions may facilitate the establishment of tumouroids from very well-differentiated tumours remains to be determined.

In a parallel study, Nuciforo and colleagues^92^ elegantly showed that long-term organoid cultures can be established from fine needle biopsies from patients with HCC. These organoids retained the morphology, expression pattern and genetic heterogeneity of the originating tumours. Similarly, PLC tumouroids have also recently been established from 91 mouse liver tumour tissues, in what represents the largest collection of liver tumour organoids ever reported. In this study, Cao and colleagues^93^ successfully used the generated organoids to assess anticancer drug responses, showing again that liver cancer organoids recapitulate the heterogeneous therapeutic responses that are observed in patients.

Of note, PLC, in particular CC, is characterised by a high degree of stromal reaction and desmoplasia. Unfortunately, all PLC tumour organoid cultures developed until now have only focused on expanding the epithelial counterpart of the tumour, and whether other tumour cell types can be incorporated in the structures and recapitulate the histopathological characteristics of the tumours in the dish is still to be investigated. In that regard, pancreas cancer organoids established by Boj *et al* 32 have recently been cocultured with stromal cells,^94^ yet in this report the cells showed little cell–cell contact and were mainly segregated in the well, with the stromal cells adhering to the bottom of the plate. Whether coculturing epithelial and non-epithelial tumour tissue together could give rise to a structural architecture that would recapitulate the tumour of origin and the severe desmoplasia present in these tumours still remains to be determined.

##### Liver infections

The liver is the target organ of many viruses and of the *Plasmodium* parasite, the agent causing malaria. Research has relied heavily on human hepatoma cell lines and humanised mouse models, but these are poor systems to recapitulate the complex biology of hepatocytes, difficult to obtain and highly costly, and not practical for large drug screening purposes.^95^ Liver organoid cultures recapitulate to some extent the complexity and architecture of the liver and may offer novel insights into the interactions between the host and *Plasmodium*. In that regard, recent studies using iPSC-derived liver organoids have shown how these are a suitable in vitro culture system to study and model HBV^96^ and HCV^97^ infections. Differentiated liver organoids retain the innate immune responses and maintain cell polarity of hepatocytes, recapitulating the natural entry of HBV and HCV and allowing their cell-to-cell transmission. While the 3D liver organoids recapitulate host–virus interactions more faithfully than the standard in vitro models to date, whether they can be used for screening antiviral treatments or propagating the virus in culture awaits further investigation.

### Regenerative medicine

Currently orthotopic liver transplantation is the only effective treatment for end-stage hepatic failure.^98^ However, a shortage of healthy tissue suitable for transplantation makes it impractical to implement as a routine therapy. Moreover, the patient receiving the transplant often needs to undergo long-term immunosuppression therapies.^99^ The expansion and differentiation potential of liver organoids makes these an alternative source of functionally mature and easily expandable cells for transplantation, overcoming the current limitations. Huch and colleagues^16^ provided the first evidence for the potential of bile duct-derived organoids as a cell therapy, first with mice and later with human-derived organoids.^27^ After in vitro expansion and differentiation towards the hepatocyte fate, mouse cells were successfully transplanted into a fumarylacetoacetate hydrolase *(FAH)^−/−^* mutant mice, a mouse model of hereditary tyrosinemia type I.^16^ Lack of FAH leads to liver failure, unless the mice are administered NTBC (2-(2-nitro-4-trifluoromethylbenzoyl)-1,3-cyclohexanedione). Although FAH-positive clusters occupied a modest 1% of the liver mass, the transplant significantly increased the survival of engrafted mice compared with control mice, serving as a proof-of-principle for the use of in vitro liver organoids for cell therapy. Similar results were obtained following transplantation of human bile duct-derived organoids into mice.^27^ While groundbreaking, the differentiation efficiency from ductal cells into hepatocytes was limited. Recently, and as described above, two studies have established a second generation of hepatic organoids (hep-orgs) using primary hepatocytes. Both hep-orgs presented high engraftment capacity.^28 29^ Mouse hep-orgs exhibited an 80% engraftment of the mouse liver parenchyma when harvested at 103 days post-transplantation,^29^ a marked improvement over the previous engraftment rate seen with ductal organoids.^27^ Similarly, human embryonic hep-orgs also presented a superior engraftment ability with 10 µg/ml of albumin secreted to the mouse bloodstream at day 45 after transplantation^28^ compared with 100 ng/ml of protein reported from ductal organoids.^27^

Future studies are needed to identify the signals driving the proliferation of transplanted hepatocyte organoids. Whether hep-orgs maintain their proliferating and engraftment capacity when transplanted into more clinical settings, namely chronically damaged livers marked by inflammation and fibrosis, will require further investigation. In these cases, how a proinflammatory and/or fibrotic environment may affect the ability of the transplanted cell to proliferate and engraft might represent a big challenge ahead. A modification of Huch *et al*’s original protocol^27^ allowed Sampaziotis and colleagues^100^ to isolate and propagate human cholangiocytes from the extrahepatic biliary tree. Theses biliary organoids were able to reconstruct the gallbladder wall and repair the biliary epithelium following transplantation into the mouse. These studies represent a major step forward in our ability to expand functional hepatocytes and cholangiocytes for therapeutic applications.

### Drug discovery and personalised medicine

The disease modelling capacity of liver organoids, either generated from adult stem cells or iPSCs (box 1), has opened the door for liver organoids as a platform for drug screening and toxicology tests.

Box 1 **Liver organoids as liver disease modelling tools: advantages and current limitations**

**Advantages.**

- Three-dimensional spatial organisation (ASCs/iPSCs).
- Genetically stable (ASCs).
- Preservation of genetic and epigenetic signature of derived tissue (ASCs).
- Long-term culture (ASCs/iPSCs).
- Biobanks (ASCs/iPSCs).
- Safe for transplantation (ASCs).
- Unlimited source of patient-derived cells (iPSCs).
- Non-invasive derivation from a variety of cells (eg, skin/fibroblasts/blood cells) (iPSCs).
- Recapitulate different aspects of liver development (iPSCs).
- Disease modelling (ASCs/iPSCs).
- High-throughput drug screening (ASCs/iPSCs).
- Personalised medicine (ASCs/iPSCs).
- Gene therapy (ASCs/iPSCs).

**Current limitations.**

- Persistence of fetal markers (iPSCs).
- Limited cell maturation (ASCs/iPSCs).
- Restricted access to tissue and need for invasive methods (ASCs).
- Failure to recapitulate the multiple cell types of the liver (ASCs).

ASCs, adult stem cells; iPSCs, induced pluripotent stem cells.

As previously discussed, using PLC tumouroids researchers were able to identify, on a small scale, the tumour’s sensitivity to different therapies,^73^ a proof-of-principle that liver organoids might be applicable to patient-tailored treatments. Along these lines, an emerging application for liver organoids is the creation of biobanks that can be used as screening platforms to identify drug efficacy against PLCs and potentially other liver diseases. Liver organoid biobanks can also be established from healthy liver cells and used as a platform for predicting drug-induced liver injury (DILI), the main reason for acute liver failure and the primary cause of drug removal from the market.^101^ While human primary hepatocytes are currently the ‘gold-standard’ for drug metabolism and toxicity testing, their availability and long-term culture are limited. The newly established human embryonic hep-org represents a potential step forward for the pharmaceutical industry in their quest to assess new therapies for potential DILI. In that regard, whether embryonic human liver organoids develop to adult stages or whether mouse adult organoids recapitulate their human counterparts and express all the metabolising enzymes required to assess human DILI remains to be investigated.

## Conclusions and future directions

While liver organoids recapitulate key aspects of the liver (eg. architecture, certain functions and genetic signature) their use in biomedical applications on a large scale is still limited by our current inability to control organoid size, shape and cell composition (box 1). Use of liver organoids as a potential strategy for regenerative medicine is dependent on their reproducibility, scalability and safety as much as their cost-effectiveness. Bioengineering holds great promise to generate liver organoids that are more physiologically relevant but also more amenable to biomedical applications.^19^

Harnessing the potential of liver cells to generate liver organoids has fuelled our ability to recapitulate in vitro the epithelial cells of the liver. However, disease modelling of complex diseases, such as PLCs where the microenvironment is key, calls for the generation of multicellular liver organoids where the epithelial cells interact with endothelial, mesenchymal and immune cells. Working closely with bioengineers to incorporate blood vessels into liver organoids could be seen as a must, and clearly a potential strategy to address the limited nutrient availability that ultimately comes into play when growing organoids.

The potential of liver organoids as tools to model other key chronic liver diseases, namely NAFLD and liver fibrosis, is a promising but yet unexplored field. NAFLD has taken the lead as the most common chronic liver disease in developed countries,^102^ encompassing a broad and progressive spectrum of liver histological alterations—from fatty liver (steatosis) to non-alcoholic steatohepatitis (NASH)—which is an established risk factor for both HCC^103 104^ and CC.^105^ An additional strong predictor of liver-related mortality risk in NAFLD^106^ is fibrosis (an abnormal although reversible deposition of ECM proteins). Despite its high prevalence, little is known about the mechanisms underlying NAFLD and its progression into NASH in the human liver. A recent report from Takebe’s lab has shown that iPSCs differentiated into hepatocyte organoids recapitulate some aspects of NAFLD and NASH in vitro, namely lipid accumulation and fibrosis, on treatment with free fatty acids.^107^ This system holds the potential to be used as a platform for drug testing and identification of potential new therapeutics for this highly prevalent disease. However, whether these iPSC-derived hepatocyte organoids can recapitulate the progressive nature of NAFLD to NASH, and subsequent step from NASH to cirrhosis and HCC, which in patients requires years, still awaits further investigation. Transplantation of these organoids into mice might facilitate that progression. Whether patient-derived liver organoids, if proven able to recapitulate the features of the disease, may help find biomarkers for risk progression into NASH and infer patient-tailored therapeutics remains to be studied.

Several liver diseases, including liver cancer, experience significant epigenetic changes that modify the epigenetic landscape of the liver cells.^108^ Whether adult tissue-derived liver organoids and/or iPSC-derived 3D liver organoids maintain the epigenetic signature of these diseases and whether they can be used to investigate the role played by epigenetic modifiers in disease initiation and progression remains to be determined. In that regard, engineered *BAP1* mutations in healthy liver organoids have recently enabled the identification of the role of the epigenetic modifier BAP1 in CC.^109^ In this study, BAP1, a predicted histone deubiquitinase, was found to control epithelial integrity through the regulation of chromatin accessibility, which results in an acquisition of malignant features in organoids already harbouring other CC mutations.^109^ Similarly, postnatal tissue-derived intestinal organoids have been shown to retain the epigenetic landscape (methylome) of the tissue of origin.^110^

In conclusion, liver organoids, whether derived from PSCs, embryonic or adult healthy or diseased tissue, are providing excellent opportunities to study human liver in an unprecedented manner. Parallel to furthering our fundamental understanding of liver development, biology and disease, human liver organoids excel as promising tools for a wide range of biomedical applications, from disease modelling of rare disorders to personalised medicine or cell therapy. This exciting challenge will require a multidisciplinary approach, with biologists, clinicians and bioengineers working closely to further understand how liver cells can self-organise to build the liver, one of the most complex organs in our body.
